# There is life beyond the statistical significance

**DOI:** 10.1186/s12978-021-01131-w

**Published:** 2021-04-17

**Authors:** Agustín Ciapponi, José M. Belizán, Gilda Piaggio, Sanni Yaya

**Affiliations:** 1 Instituto de Efectividad Clínica Y Sanitaria (IECS-CONICET). Cochrane Argentina, Emilio Ravignani 2024, C1414CPV Buenos Aires, Argentina; 2 Statistika Consultoria, São Paulo, Brazil; 3 Reproductive Health, Buenos Aires, Argentina; 4 School of International Development and Global Studies, University of Ottawa, Ottawa, ON Canada; 5 The George Institute for Global Health, Imperial College London, London, UK

## Abstract

This article challenges the “tyranny of P-value” and promote more valuable and applicable interpretations of the results of research on health care delivery. We provide here solid arguments to retire statistical significance as the unique way to interpret results, after presenting the current state of the debate inside the scientific community. Instead, we promote reporting the much more informative confidence intervals and eventually adding exact P-values. We also provide some clues to integrate statistical and clinical significance by referring to minimal important differences and integrating the effect size of an intervention and the certainty of evidence ideally using the GRADE approach. We have argued against interpreting or reporting results as statistically significant or statistically non-significant. We recommend showing important clinical benefits with their confidence intervals in cases of point estimates compatible with results benefits and even important harms. It seems fair to report the point estimate and the more likely values along with a very clear statement of the implications of extremes of the intervals. We recommend drawing conclusions, considering the multiple factors besides P-values such as certainty of the evidence for each outcome, net benefit, economic considerations and values and preferences. We use several examples and figures to illustrate different scenarios and further suggest a wording to standardize the reporting. Several statistical measures have a role in the scientific communication of studies, but it is time to understand that there is life beyond the statistical significance. There is a great opportunity for improvement towards a more complete interpretation and to a more standardized reporting.

## The debate

For decades, the “P-value”-based binary interpretation and reporting of results, based on a cutoff point for the P-value dominated the publications. However, many concerns arose about such rules dictating clinical implications of research results. Nowadays, the scientific community seems to agree that this binary approach is not advisable. The problem is that treating results as either ‘statistically significant’ or ‘statistically non-significant’ implies categorizing a continuous variable, which is at least misleading [1, 2].

## Is there a rationale for a change?

In a very influential publication in “Nature”, Valentin Amrhein and more than 800 scientists including statisticians, clinical and medical researchers, biologists and psychologists worldwide rose up against statistical significance and its misleading interpretation [3]. They claim against this limited dual point of view as opposed to making use of life’s profuse palette of colors. Moreover, they highlight that a statistically non-significant result does not prove the null hypothesis [4]. Nevertheless, this is a prevalent practice since 51% (402/791) articles from five journals erroneously interpret statistically non-significant results as indicating “no effect” [3]. In the same way, it would be inappropriate to conclude that an association or effect inexorably exists just because it was statistically significant. Besides, two studies reporting P-values lower or higher than 0.05 are not necessarily in conflict considering that the point estimate could be exactly the same and the lack of statistical power of one of the studies could explain this difference. In other words, to draw conclusions of scientific, clinical or practical importance based only on statistical significance is not recommended. The binary interpretation approach has had such a deep impact among journal editors that it contributed to publication bias by considering as unworthy the studies with non-significant results. In this context, the proportion of statistically significant estimates is usually biased upwards. On the other hand, a result with high statistical significance (e.g.: P < 0.000001) only implies that the observed finding has a very low probability of occurring by chance but that it could perfectly be clinically irrelevant.

This debate is not new [5, 6], however, there are still several discussions with journal editors and reviewers regarding how to report or interpret manuscripts results. In the editorial special issue of The American Statistician about this topic, authors stated that we need to move to a world beyond “P < 0.05” [7]. These authors recognize that statements and position papers addressing this need for a variety of audiences were important but insufficient to reach a cultural change. The title of one study of this special issue amusingly illustrates this concept: “the difference between ‘significant’ and ‘not significant’ is not itself statistically significant” [8]. Meta-research strongly highlighted the adverse effects of misinterpretation of *P-value*s and significance judgements in individual studies.

## What should not be done?

However, given that “statistical significance” is so frequently misreported and misinterpreted, there seems to be a consensus that terms such as ‘significant’, ‘statistically significant’, ‘borderline significant’, and their negative expressions, should not be reported anymore. The expression ‘trend towards’ is also frequently misused as “near statistically significant” and prone to bias if used selectively. It is invalid to only report the trends aligned with the researcher’s hypothesis, but the approach is acceptable if all trends, regardless of their direction, are reported. Furthermore, a P < 0.05 should not be considered at all when deciding which results to present or highlight. It is correct to highlight significant results, but the decision on what result to present or highlight should be based on the research hypothesis not on which result is significant. Similarly, Ioannidis recommends not to use P-values unless there is a clear justification that it is the best choice, and to be always highly skeptical about “statistically significant” results at the 0.05 level [9].

Reporting and interpreting point and interval estimates together should be the rule but it has not been the case for a long time. The number of studies using the term “statistically significant” but not mentioning confidence intervals (CIs) for reporting comparisons in abstracts range from 18 to 41% in Cochrane Library and in the top-five general medical journals between 2004 and 2014 [10]. These findings were even worse for other topics like infertility journals [11]. There were clear recommendations to overcome this problem in the Cochrane Handbook since September 2008, but there is a lack of such recommendations in other journals.

## What should be done to integrate statistical and clinical significance?

P-values must still be used, but they should be reported as continuous exact numbers (e.g., P = 0.07), clearly describing its scientific or practical implications to better interpret it. Moreover, rather than adopting rigid rules for presenting and interpreting continuous P-values, we need a case by case thoughtful interpretation considering other factors such as certainty of the evidence, plausibility of mechanism, study design, data quality, and costs-benefits that determine what effects are clinically or scientifically important. It is also important to remember that clinical implications of results cannot be extrapolated to patient groups other than the patients included in a study [12].

There are many frequentist and Bayesian tools to provide a significance level [5], but the P-value should be interpreted in the light of its context of sample size and meaningful effect size. Thus, we need to distinguish between statistical and clinical significance. Although we will use the term "clinical significance" in this text, it may be a good idea to replace it with "clinical relevance" and to apply the term “significance” only to statistical issues. For example, a two-stage approach to inference requires both a small P-value and a pre-specified sufficiently large effect size to declare a result “significant” [13]. Predetermining whether an effect size is relevant for the patient is much more important than the statistical significance [14]. This is the minimal important difference (MID) that is the smallest change in a treatment outcome that an individual patient would identify as important and that would indicate a change in the patient's management. This term is preferred to the minimal clinically important difference (MCID) because this terminology focuses attention on the clinical aspects rather than patients' experience [15, 16] and should be presented with the minimum and maximum of the scale and its direction [17] to facilitate the interpretation of results [10, 13]. This term is generally applied to continuous outcomes, but it could be used for other types of outcomes as well.

Deciding whether the size of an effect is relevant or not depends on how critical an outcome is. For example, it is difficult to define a lower threshold for clinical significance/relevance of mortality estimates as of any benefit of a new treatment, whatever small, is relevant [12, 18]. Conversely, the lower limit threshold will necessarily be higher for the less important outcomes. Thus, the threshold should be based on how much the intended beneficiaries value each relevant outcome and what they would consider to be an important absolute effect. There are several recommended methods for determining MID for patient-reported outcomes [19]. However, the information on how much people value the main outcomes varies and is unreliable. In this case, the authors should at least state that the MID will be based on their own judgement [20].

A judgment and rationale are required to decide what constitutes appreciable benefits and harms. Regardless of the type of outcome, an intervention with a small beneficial clinically relevant effect will not be recommended if adverse effects are relevant [12, 18]. Serious adverse effects, even if rare, may make the use of an otherwise beneficial intervention not justified. Therefore, it is mandatory to assess the harmful effects to determine the clinical significance of an intervention [12, 18]. Moreover, if a new intervention is classified as having “statistically significant” effects but its effect size is smaller compared to another intervention, then the new intervention effect might be considered as “not clinically significant”. Hence, the effect size of alternative interventions for a condition could help to state clinical significance thresholds for an intervention of interest. This threshold should focus on both relative and absolute effects, since it is difficult, if not impossible, to judge the importance of a relative effect alone. For example, a relative risk reduction of 20% for women with a 20% likelihood of abortion would mean a risk difference (an absolute effect) of 4%, or a Number Needed to Treat to Benefit (NNTB) of 25. However, the same relative effect for women with a 1% likelihood of abortion would mean an absolute risk difference of only 0.2% or an NNTB of 500, which represent a much less important effect.

For a drug with no serious adverse effects, minimal inconvenience, and modest cost, even a small effect would warrant a strong recommendation. For instance, we may strongly recommend an intervention with a MID of at least 0.5% of absolute risk reduction of abortion (NNTB of 200). However, if the treatment is associated with serious toxicity, we could prefer a more demanding MID like 1% (NNTB of 100).

We consider that reporting a point estimate and CI is much more informative and should be the rule. Additionally, the P-value informs the probability that this effect has been observed by chance.^1^ Therefore, it is much better to report the exact P-value than the binary approach of statistical significance based only on the arbitrary cut-off point of 0.05.

However, a binary use of confidence or credible intervals (focused on whether such intervals include or exclude the null value) could lead to the same problem caused by the use of the statistical significance. In fact, some authors propose the alternative term “compatibility” intervals to guard against overconfidence [21]. Authors should describe the practical implications of all values inside the interval compatible with the data, especially the observed effect (or point estimate) that is the most probable or compatible result of this interval. Even with large P-values or wide intervals, authors should discuss the point estimate, as well as discussing the limits of that interval. An interval containing the null value will often also contain non-null values of high practical importance that should not be left out of the conclusions. If the imprecision is accepted and properly interpreted, we will embrace replications and the integration of evidence through meta-analyses, which will in turn give us more precise overall estimates.

**Fig. 1:**
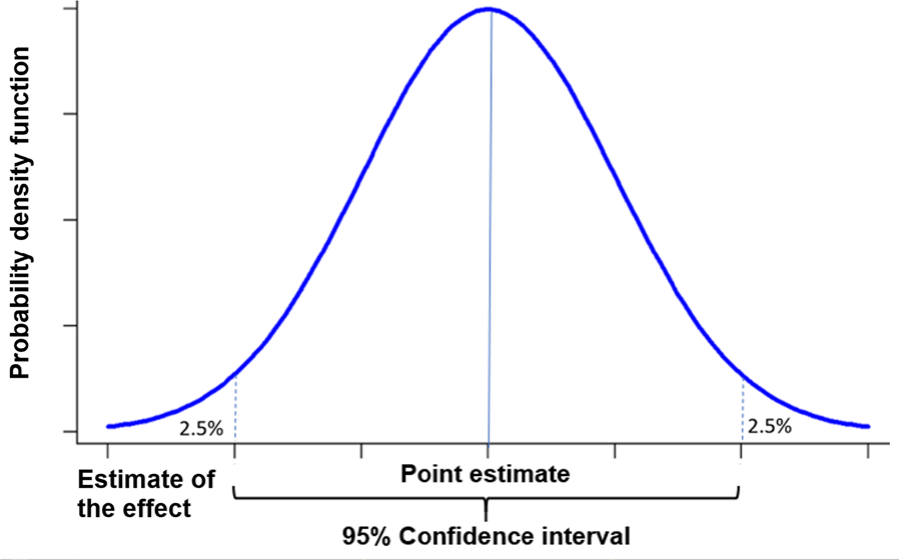
Probability density function of the difference between two sample means. The point estimate is the most likely value of the parameter of interest across the CI. A confidence interval (CI) is a range of values used to estimate a population parameter and is associated with a specific confidence. With a CI of 95% confidence, there is a 95% probability that any given CI will contain the true population parameter and a 5% chance that it won’t (two tails of 2.5%)

One of the most polemic scenarios is the situation of point estimate showing important clinical benefits with 95% CIs compatible with both, even better benefits or important harms and a P-value > 0.05 (i.e. P = 0.08). We have argued against interpreting and/or reporting results as statistically non-significant. A better statement could be “the intervention did not demonstrate superiority vs the comparator”. Although this is true, it is still possible that this result could be interpreted as indicating “no effect” [3] and this is not outside of the binary logic of superiority. On the other hand, it is possible to report that the intervention might be superior to its comparator, but it is also compatible with beneficial or detrimental effects. The supporters of the “non-superiority statement” claim against the last option because it could be misinterpreted as a positive effect. However, the P-value of this example indicates that the chance of concluding that there is a difference where, in reality, none exists (Type I error or false positive). This proportion could be unacceptable for conclusions, but it is not so high in terms of probability. Additionally, the estimate of the effect that has the maximum likelihood across the CI is the point estimate. To illustrate this point, consider an example in which the effect to be estimated is the difference between two means of normally distributed populations. Two independent samples from these populations yield sample means, and their difference with a 95% confidence interval has been calculated. Figure 1 presents the probability density function of this difference (with the 95% confidence interval for the effect indicated). In real life, distributions are likely to deviate from normality and the confidence interval for an effect might not be symmetric around the point estimate.

Therefore, it seems fairer to report the point estimate as to the more likely value jointly with a very clear statement of the implications of extremes of the confidence interval. In fact, this is the approach recommended in the Grading of Recommendations Assessment, Development, and Evaluation (GRADE) guidelines [22].

## What is the key role of the minimum important difference (MID) in the interpretation of the results?

**Fig. 2:**
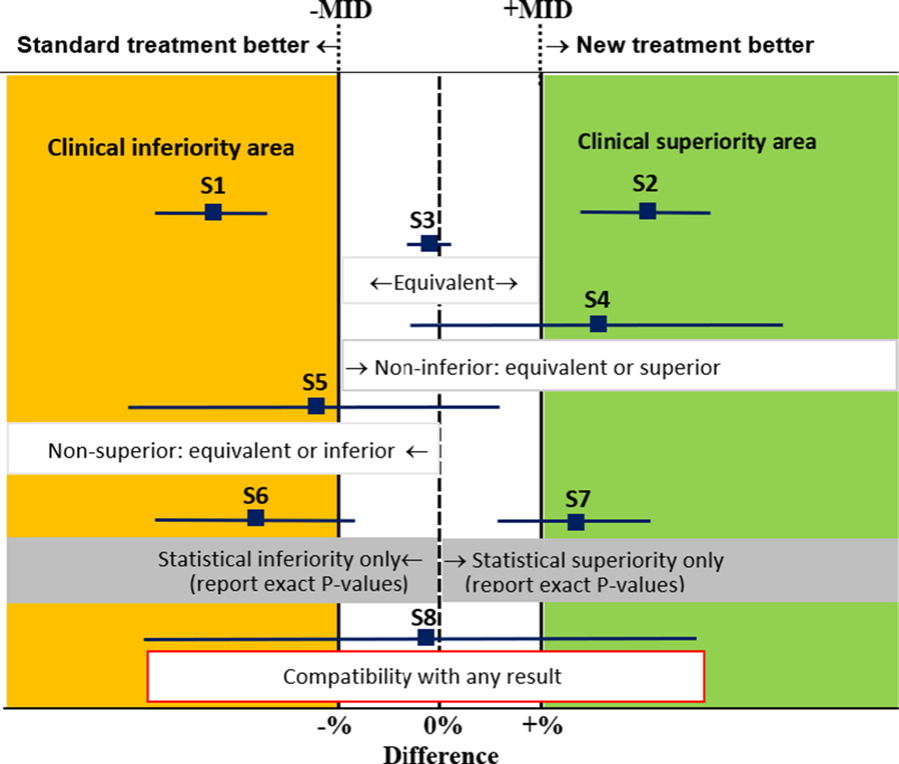
Interpretation of results for different scenarios according to statistical and clinical thresholds or minimal important difference (MID). Minimal (clinically) important difference (MID). The blue squares indicate the point estimate of the effect of a new treatment compared with a standard treatment, and the blue lines on either side of it the 95% confidence interval

To better explain these concepts, Fig. 2 shows different scenarios of individual study results (point estimate and 95% CIs) in relation to the MID, for the difference of proportions of a desirable outcome between a New minus a Standard treatment. The interpretation of these scenarios varies depending on statistical and clinical thresholds (which are not necessarily the same). The clinical (or practical) threshold, denoted as MID, defines the cut-off points for clinical superiority and inferiority areas in this graphic. The limit beyond which a difference can be considered as clinically significant (superior or inferior) is mainly based on the balance of desirable and undesirable effects, economic considerations for individuals and health systems and values and preferences. In other words, this limit has serious implications for decision-making and interpretation of the results. An effect will be considered clinically important or not, depending on whether it's 95% CI crosses this threshold.

For example, the Study 1 (S1) is completely inside of the clinical inferiority area for the New treatment since its upper 95% CI limit is below -MID and the related P-value of 0.0001 indicates that the probability that the cause of the observed difference could be attributed by chance is only 1/10,000. A mirror scenario in the opposite direction occurs with S2 completely inside of the clinical superiority area for the New treatment.

Another lesson is that “statistically non-significant results may or may not be inconclusive” even with the same point estimate. For example, S3 presents a very narrow 95% CI and a P-value of 0.70 and is conclusively considered as “Equivalent”. S8 presents a very wide 95% CI and a P-value of 0.90 and its results are inconclusive since it is compatible with both important benefits and harms.

S4 could only be considered clinically as “non-inferior” and statistically non-significant (P = 0.06, wide 95% CI crossing the null effect line but not the clinical inferiority line). S2 and S4 present similar point estimates but S2 presents a narrower 95% CI inside de clinical superiority area and P = 0.01).

## Are there reporting guidelines?

To express narratively the aforementioned concepts in order to effectively, and consistently, communicate the conclusions of a review is even more challenging [23]. Cochrane has recently adopted an approach based on the integration of the effect size and certainty of evidence classified by the GRADE approach (see Table 1) [24].

**Table 1:** Suggested narrative statements for phrasing conclusions

Certainty of the evidence	Effect size	Suggested statements for conclusions (replace X with intervention, choose ‘reduce’ or ‘increase’ depending on the direction of the effect, replace ‘outcome’ with name of outcome, include ‘when compared with Y’ when needed)
High	Large	X results in a large reduction/increase in outcome
	Moderate	X reduces/increases outcome
		X results in a reduction/increase in outcome
	Small important effect	X reduces/increases outcome slightly X results in a slight reduction/increase in outcome
	Unimportant or no effect	X results in little to no difference in outcome X does not reduce/increase outcome
Moderate	Large	X likely results in a large reduction/increase in outcome X probably results in a large reduction/increase in outcome
	Moderate	X likely reduces/increases outcome X probably reduces/increases outcome X likely results in a reduction/increase in outcome X probably results in a reduction/increase in outcome
	Small important effect	X probably reduces/increases outcome slightly
		X likely reduces/increases outcome slightly
		X probably results in a slight reduction/increase in outcome
		X likely results in a slight reduction/increase in
	Unimportant or no effect	X likely results in little to no difference in outcome X probably results in little to no difference in outcome X likely does not reduce/increase outcome X probably does not reduce/increase outcome
Low	Large	X may result in a large reduction/increase in outcome
		The evidence suggests X results in a large reduction/increase in outcome
	Moderate	X may reduce/increase outcome The evidence suggests X reduces/increases outcome X may result in a reduction/increase in outcome The evidence suggests X results in a reduction/increase in outcome
	Small important effect	X may reduce/increase outcome slightly The evidence suggests X reduces/increases outcome slightly X may result in a slight reduction/increase in outcome The evidence suggests X results in a slight reduction/increase in outcome
	Unimportant or no effect	X may result in little to no difference in outcome The evidence suggests that X results in little to no difference in outcome X may not reduce/increase outcome The evidence suggests that X does not reduce/increase outcome
Very low	Any effect	The evidence is very uncertain about the effect of X on outcome X may reduce/increase/have little to no effect on outcome but the evidence is very uncertain

GRADE defines evidence certainty (previously quality) differently for systematic reviews and guidelines. For systematic reviews, certainty mainly refers to the confidence in the estimates of effect for a specific outcome. For guidelines, certainty refers to the extent to which our confidence in the effect estimate is adequate to support a particular decision.

As the certainty of the evidence incorporates a judgement about the precision of effect estimates, it is not necessary to say anything more about the confidence interval or P-value in these statements about effects. However, situations where the point estimate indicates an important benefit (S4 and S7) or harm (S5 and S6), but the CIs are wide involving no benefit or harm, regardless of the non-statistical significance (like S4 and S5 cases) should be explained using statement mentioning both possibilities:

[Intervention] may lead to [better outcome (S4, S7)/worse outcome (S5, S6)], however, the 95% CI indicates that [intervention] might make little or no difference/might worsen/increase [outcome].

[Intervention] may lead to [little to no difference in outcome (S8)], however, the 95% CI is compatible with both important beneficial and harmful effects.

For example, “Using lay health workers as an add-on to usual care may increase care-seeking behavior for children under five. However, the 95% confidence interval indicates that it might make little or no difference” [20].

GRADE guidelines suggest a default threshold for appreciable relative benefit and harm of 25% ± 5% [25, 26] if there is no clear evidence to establish a more rational cut-off point. Other groups like the Institute for Quality and Efficiency in Health Care (IQWiG) use increasing relative differences or thresholds for determining both the effect size of interventions (major, considerable and minor), and related to the importance of the outcomes considered (all-cause mortality, serious symptoms and adverse events, as well as health-related quality of life, and non-serious symptoms and adverse events) [27].

Having determined a threshold for an important effect, it is highly desirable to make it explicit in the protocol of any study assessing interventions.

Preparing summary of findings (SoF) tables for systematic reviews according to the GRADE guidelines [22] (the current standard to grade the certainty of evidence) is an excellent way to demonstrate the clinical implications of the results. Reporting confidence intervals, ‘minimal clinical relevant differences’ for continuous outcomes, numbers-needed to-treat for binary outcomes, and median survival times for survival data may also improve the clinical interpretability of results.

The main recommendations that emerge from these considerations are:

**Table:** 

1. Include confidence intervals (and exact P-values when relevant) but do not report results as being statistically significant or non-significant 2. Refer to a minimal important difference, as soundly as possible, to establish clinical or practical significance 3. Present the effect estimates for each outcome together with the certainty of the evidence of the effect (high, moderate, low, and very low) 4. To draw conclusions, consider the multiple factors besides P-values such as certainty of evidence, net benefit, economic considerations and values and preferences 5. Present the results consistently, using similar words and expressions, such as those suggested in Table 1, for similar effects sizes and certainty of the evidence	

## How to access life beyond the statistical significance?

A reform is necessary for moving beyond statistical significance but, considering the barriers to change, maybe it would be better to define it as a revolution. Goodman says that the explanation is neither philosophical nor scientific, but sociologic: “When everyone believes in something’s value, we can use it for real things; money for food, and P-values for knowledge claims, publication, funding, and promotion. It doesn’t matter if the P-value doesn’t mean what people think it means; it becomes valuable because of what it buys” [28].

Journals, academic institutions, scientific lay and professional media (including social media), funding agencies and regulators should reduce the impact of statistical significance on publication, funding and promotion. Some groups have eliminated the binary statistical significance approach from their editorial practice.

Furthermore, the certainty of the evidence should be presented together with effect estimates for each outcome using similar words and expressions, such as those suggested in Table 1, for similar levels of importance of the effects and certainty of the evidence. This standard wording should replace alternative wordings that are frequently ambiguous or even incorrect.

Changing editorial policies will not be easy to implement, but if journals provide clear guidelines to authors, besides improving the quality of reporting, the editorial work burden will be reduced at the end of the day. The CONSORT (CONsolidated Standards of Reporting Trials) 2010 guidelines for reporting parallel group randomized trials encourage the use of continuous exact P-values alongside with CIs, but it does not include a strong position against the binary way of reporting results [29]. Surely an updated version of the CONSORT statement and continuous statistics education could be beacons for improving the reporting of studies.

A detailed statistical analysis plan preventing the misuse of the statistical significance should also be a request to approve a protocol that must be accessible to other researchers. It should be based on strong theory and a context of relevant prior empirical evidence. Therefore, the study team should have statistical knowledge, skills and experience to interpret and communicate findings.

Regulatory agencies like the U.S. Food and Drug Administration (FDA), have long-established significance thresholds for P-values for Phase III drug trials. This kind of institutions might require more time for making changes. The objection against retiring statistical significance is that it is needed to make yes-or-no decisions, however this misconception ignores that decisions are made based on multiple factors besides P-values including net benefit, economic considerations and values and preferences.

The life beyond the statistical significance will emphasize estimates and the uncertainty around them which will be accompanied by a discussion about the practical implications of the lower and upper limits of their intervals in the context of MIDs. Hopefully, the methods sections will be more detailed and anchored in real life. They will provide more background information that allows other researchers to execute meaningful alternative analyses. Thus, the interpretation of results not based on statistical thresholds will promote thinking about the potential consequences of the estimations and preventing confusions between statistics and reality.

## Conclusions

The “P-value”-based binary interpretation and reporting of results is insufficient to capture the complexity of the problem and limits the true understanding of the evidence. There is a great opportunity for improvement towards a more complete interpretation and hopefully to a more standardized reporting.

The statistical community has not yet reached a consensus about the interpretation of statistical inference for treatment effects or a wording for reporting results. But solid bases for the use of statistics and communication skills do exist, and applying them could help to greatly improve science and decision-making.

All of those who embarked in health research have imposed a rigorous methodology on research planning, analyses and reports because of the relevance of research results for the health, quality of life and survival of the population. It is a duty to be proactive in continuously improving health research methodology. The discussion in this article is a compelling call to reconsider the “tyranny of P-value” and move towards interpretations that are more valuable with better applicability of the results of research on health care delivery. Those responsible for producing and communicating the scientific literature—authors, editors, reviewers—need to apply the considerations and recommendations set out in this commentary in the generation, evaluation and publication of articles.

## Footnotes

1 Given the null hypothesis. p-value = Pr(data | H0).
